# Into the Bowels of Depression: Unravelling Medical Symptoms Associated with Depression by Applying Machine-Learning Techniques to a Community Based Population Sample

**DOI:** 10.1371/journal.pone.0167055

**Published:** 2016-12-09

**Authors:** Joanna F. Dipnall, Julie A. Pasco, Michael Berk, Lana J. Williams, Seetal Dodd, Felice N. Jacka, Denny Meyer

**Affiliations:** 1 IMPACT Strategic Research Centre, School of Medicine, Deakin University, Geelong, VIC, Australia; 2 Department of Statistics, Data Science and Epidemiology, Swinburne University of Technology, Melbourne, Victoria, Australia; 3 Melbourne Clinical School-Western Campus, The University of Melbourne, St Albans, VIC, Australia; 4 Department of Epidemiology and Preventive Medicine, Monash University, Melbourne, VIC, Australia; 5 University Hospital Geelong, Geelong VIC Australia; 6 Department of Psychiatry, The University of Melbourne, Parkville, VIC, Australia; 7 Florey Institute of Neuroscience and Mental Health, Parkville, VIC, Australia; 8 Orygen, the National Centre of Excellence in Youth Mental Health, Parkville, VIC, Australia; 9 Centre for Adolescent Health, Murdoch Children’s Research Institute, Melbourne, Australia; 10 Black Dog Institute, Sydney, Australia; Istituto Superiore Di Sanita, ITALY

## Abstract

**Background:**

Depression is commonly comorbid with many other somatic diseases and symptoms. Identification of individuals in clusters with comorbid symptoms may reveal new pathophysiological mechanisms and treatment targets. The aim of this research was to combine machine-learning (ML) algorithms with traditional regression techniques by utilising self-reported medical symptoms to identify and describe clusters of individuals with increased rates of depression from a large cross-sectional community based population epidemiological study.

**Methods:**

A multi-staged methodology utilising ML and traditional statistical techniques was performed using the community based population National Health and Nutrition Examination Study (2009–2010) (N = 3,922). A Self-organised Mapping (SOM) ML algorithm, combined with hierarchical clustering, was performed to create participant clusters based on 68 medical symptoms. Binary logistic regression, controlling for sociodemographic confounders, was used to then identify the key clusters of participants with higher levels of depression (PHQ-9≥10, n = 377). Finally, a Multiple Additive Regression Tree boosted ML algorithm was run to identify the important medical symptoms for each key cluster within 17 broad categories: heart, liver, thyroid, respiratory, diabetes, arthritis, fractures and osteoporosis, skeletal pain, blood pressure, blood transfusion, cholesterol, vision, hearing, psoriasis, weight, bowels and urinary.

**Results:**

Five clusters of participants, based on medical symptoms, were identified to have significantly increased rates of depression compared to the cluster with the lowest rate: odds ratios ranged from 2.24 (95% CI 1.56, 3.24) to 6.33 (95% CI 1.67, 24.02). The ML boosted regression algorithm identified three key medical condition categories as being significantly more common in these clusters: bowel, pain and urinary symptoms. Bowel-related symptoms was found to dominate the relative importance of symptoms within the five key clusters.

**Conclusion:**

This methodology shows promise for the identification of conditions in general populations and supports the current focus on the potential importance of bowel symptoms and the gut in mental health research.

## Introduction

Depression is a debilitating illness that is estimated to affect 350 million people globally and is frequently associated with somatic symptoms and other medical conditions [1,2]. The nature and direction of these relationships are often complex, interrelated, and difficult to unravel. Depression classically presents with many and diverse somatic symptoms. The comorbidity of depression with a number of chronic medical conditions, such as Irritable Bowel Syndrome (IBS) [3], ischemic heart disease [4], cancer [5], diabetes [6], osteoporosis [7], thyroid disease [8], and obesity [9], has also been well established. However, these conditions often have bidirectional relationships with depression such that this level of comorbidity and interrelatedness can complicate treatment and stymie efforts to identify causal factors in depression. Thus, the identification of individuals in clusters of comorbid symptoms in depression may reveal new pathophysiological mechanisms and treatment targets.

Due to the complexity and heterogeneity of medical data, previous studies have primarily investigated individual medical conditions linked to depression. The use of “big data” and machine-learning (ML) techniques and algorithms has the ability to handle heterogeneous data without strict constraints and have been demonstrated to unearth key patterns and interactions in health data [10,11]. The mapping of multidimensional data onto two-dimensional maps [12–14] with ML techniques allows the researcher to visualise and interpret the complexity of the data and generate new hypotheses regarding depression.

ML is a vast and expanding field of artificial learning where algorithms improve performance through experiential learning [15]. In the health arena, ML algorithms that learn by training on subsets of data have been used to fit models using supervised ML (i.e. where the objective of the exercise is to establish the main inputs to predict known values) [16], and to find patterns in data using unsupervised ML (i.e. where the objective is to uncover previously unknown patterns and clusters within the data set, without any a priori model defined) [17]. Blending of unsupervised and supervised ML techniques has been used to detect patterns and relationships within large numbers of complex lifestyle-environ variables [18]. Notoriously complex in nature, medical symptom data are ideally suited to blended ML techniques. Utilising the learning properties of ML it is possible to detect, visualize and understand the composition of medical symptoms clusters for those with psychiatric disorders such as depression. [19,20]

ML techniques have been used across a variety of disciplines to explore and model very large quantities of data to discover patterns, unsuspected relationships and useful rules for a specific purpose. Often novel unsuspected and novel interpretations of the data (serendipity) are uncovered. Commercially, these techniques have been used successfully for businesses to learn from their transaction data about the behaviour of their customers, improving their business model by exploiting this knowledge [21]. However, it has only been over the last 10 years that ML techniques have been used in medical research, primarily in neuroscience and biomedicine [22,23]. More recently ML techniques have been used in psychiatry [10], using predominantly very big data sets. Complex survey methodologies are often implemented with population-based data (e.g. oversampling in underrepresented groups, stratification, clustering) and traditional statistical techniques are capable of dealing with this complexity [24]. However, big data techniques on their own do not adequately account for this type of sample. Thus, a blend of both big data ML techniques with traditional statistical techniques has the potential to uncover hidden patterns while accounting for the complex sampling.

The aim of this research was to use data from a large cross-sectional community based population epidemiological study to combine unsupervised and supervised ML algorithms with traditional regression techniques by utilising self-reported medical symptoms to identify and describe clusters of individuals with increased rates of depression from a large cross-sectional community based population epidemiological study.

## Methods

### Study design and participants

The 2009–2010 National Health and Nutrition Examination Survey (NHANES) (2009–2010) [25] cross-sectional civilian noninstitutionalized population based data were utilised for this study. This study included 18 to 80 year old non-institutionalised US civilians (N≈10,000) and applied a complex four-stage sampling methodology: counties; segments within counties; households within segments; and, individuals within households. Data were collected from 15 locations across 50 US states, with oversampling of subgroups of the population of particular public health interest, to increase the reliability and precision of population estimates [25]. Questionnaire data relating to medical symptoms and demographics were downloaded from the NHANES website and integrated using the Data Integration Protocol In Ten Steps (DIPIT) [26].

Variables were initially selected based on the criterion of relevance to medical symptoms. Analysis was performed to minimise the degree of missing data across the set of medical symptoms. The final set of 68 dichotomous medical symptom variables and an unweighted sample size of 3,922 was used for clustering in this research study. There were 377 participants identified with depression, being representative of the total depressed sample for NHANES during 2009–2010 (i.e. 8% after adjustment for the complex survey sample structure). The imbalanced nature of the data was addressed in this study by identifying clusters with high rates of depression (i.e. high risk clusters) rather than individual participants with depression. This meant that within each high risk cluster the imbalance was much reduced. This was the primary rationale for undertaking the Self-organised Mapping (SOM) and clustering of individuals, thereby allowing the identification of the key clusters significantly associated with depression using binary logistic regression. Finally, the most important medical symptoms for identifying depressed individuals were identified for each of these key clusters.

NHANES received approval from the National Center for Health Statistics (NCHS) research ethics review board and informed consent was obtained from all participants. Use of data from the NHANES 2009–2010 database is approved by the National Center for Health Statistics Research Ethics Review Board (Continuation of Protocol #2005–06).

### Study Measurements

A self-reported Patient Health Questonnaire-9 (PHQ-9) [27] was used to assess depressive symptoms (‘depression’). This questionnaire consisted of nine items that were summed to form a total score. Those with a total score of 10 or more were considered moderately or severely depressed [28]. The 68 medical symptom data were classified into 17 broad medical categories: heart, liver, thyroid, respiratory, diabetes, arthritis, fractures and osteoporosis, pain (i.e. neck, back, hip pain), blood pressure, cholesterol, vision, hearing, psoriasis, weight, bowels, urine, and if a blood transfusion was received. The self-report demographic and socio-economic variables from the NHANES demographic and questionnaire data components were also utilised [29].

### Statistical Methodology

This research implemented two ML algorithms: an unsupervised algorithm, combined with hierarchical clustering, to create the medical symptom clusters and a supervised algorithm to identify and describe the key clusters with a significant relationship with depression. Due to the complex sampling methodology of the NHANES data, traditional binary logistic regression was implemented to identify these key clusters while controlling for potential socio-demographic confounders.

**Fig 1:**
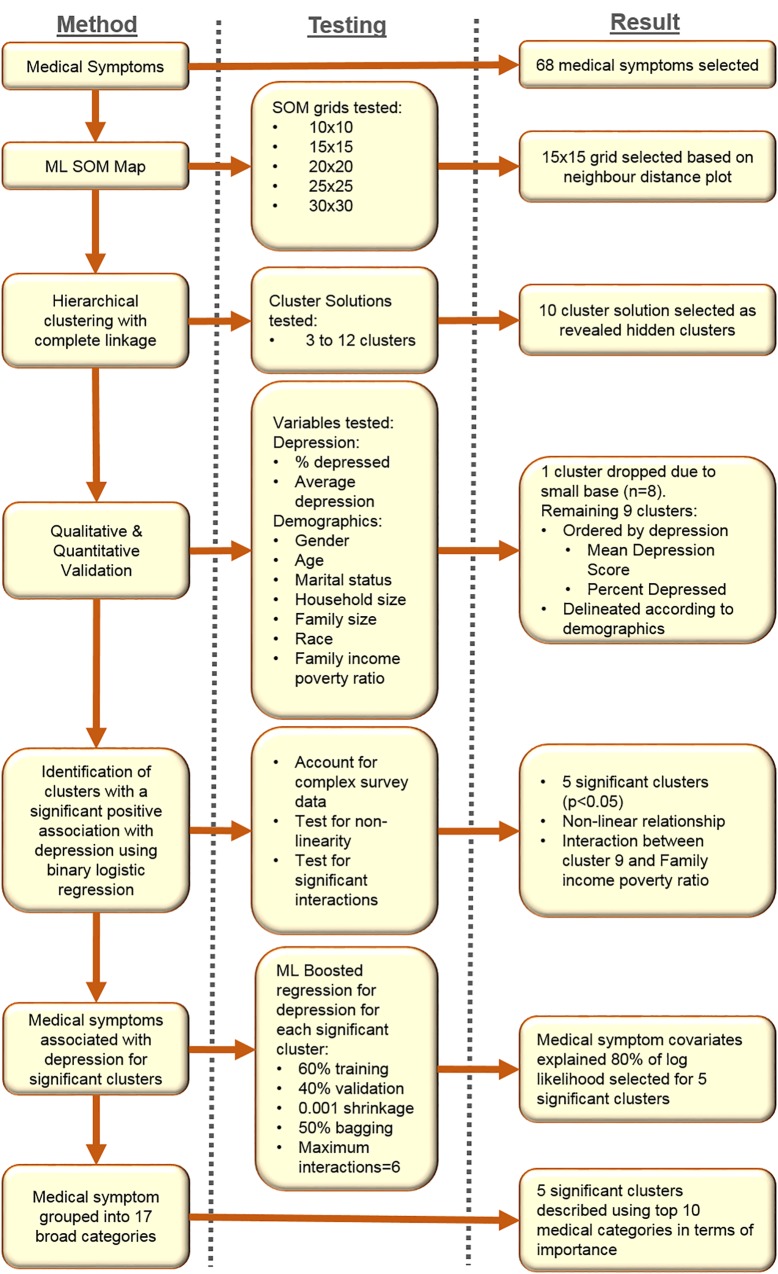
Flowchart of Methods, Testing and Results.

A summary of the statistical methodology, testing regime and results is outlined in Fig 1.

### Medical symptom cluster identification

Self-organizing maps (SOMs) were introduced by Kohonen in 1995 [30] as a variant of artificial neural networking, inspired by biological neural networks, and have since been used in many diverse applications across a variety of fields including bioinformatics, engineering, financial analysis, experimental physics, and psychiatry [31,32]. SOMs provide a simple and effective unsupervised ML algorithm for clustering individual participants and visualising high dimensional data in a low dimensional map without any reliance on distributional assumptions.

The SOM identifies clusters by effectively packing the dataset onto a q-dimensional plane where data points “similar” to each other in the original multidimensional data space are then mapped onto nearby areas of the q-dimensional output space. SOMs combine competitive learning with dimensionality reduction by smoothing the clusters with respect to an apriori grid. The SOM is called a topology-preserving map because multi-dimensional input data is represented often by a two dimensional “map” of nodes where topological properties of the input space are maintained.

The steps involved in the SOM competitive ML algorithm involve initially assigning random vector weights to each node (or position on the grid), then randomly choosing data points (participants) from the training data and presenting them to the SOM. The “Best Matching Unit” (BMU) in the map is the node with a vector weight most similar to a data point and nodes within the “neighbourhood” of each BMU are found. With each iteration, the size of this neighbourhood decreases. The vector weights of nodes in the BMU neighbourhood are adjusted closer to their associated data points. The size of these adjustments decrease with each iteration and the magnitude of these adjustments is proportional to the proximity of the node to the BMU. These steps are repeated for N iterations or until the vector weights for all the nodes converge to their final values.

For this study a hexagonal map topology was used, with five SOM grids tested (10x10, 15x15, 20x20, 25x25, 30x30) to establish a map with suitable nodes. The final solution utilised a 15x15 grid with a learning rate for weight adjustment declining linearly from 5% to 1% over 100 iterations. The unconstrained nature of the SOM technique meant that clusters of nodes form naturally from the medical symptom data on the grid without the influence of the participant’s depressive symptom status. Hierarchical clustering, using the complete linkage method [33], was then utilised to group SOM nodes with similar final weights, identifying the final clusters. Three to 12 cluster solutions were considered and the cluster solution with the most differentiation in terms of depression was chosen for further investigation. The clusters were numbered in order of their rates of depression (i.e. frequency and average total PHQ-9 score).

### Identification of key clusters with higher depression rates

Quantitative and qualitative investigation, using exploratory statistics of the resultant clusters was used to establish variation with respect to depression rates and demographics.

Demographic factors were included in a binary logistic regression model to identify the key participant clusters with a significant positive relationship with depression, accounting for the complex survey design of NHANES. This model controlled for potential confounders and quantified the probability of depression within each cluster. The cluster with the lowest depression rate was chosen as the reference group. This stage of the analysis was used to identify participant clusters with significant rates of depression in order to identify the important medical symptoms from the ML boosted regression. Only these key clusters were used in the next stage of supervised ML boosted regression. No further investigation was performed on those clusters with non-significant odds ratios for depression.

### Medical symptoms most prominent within key clusters

Supervised ML boosted regression [34], translated to a binary logistic regression analysis [35], was implemented for each of the key clusters to identify the most prominent medical symptoms associated with depression within these clusters. This technique has been previously used to identify biomarkers associated with depression [36] and to describe lifestyle clusters associated with depression [18] using data from the NHANES study. Depression was considered as a binary outcome and run for each key cluster using Friedman’s Multiple Additive Regression Trees (MART) boosted algorithm [37,38]. Consistent with previous research using this ML algorithm on the 2009 to 2010 NHANES data [36], validation was performed using a random split of each data set into 60% training and 40% validation, a regularization shrinkage parameter of 0.001, with 50% of the residuals used to fit each successive tree (50% bagging) [37]. The maximum number of boosting interactions (i.e. number of terminal nodes plus 1) allowed was six, being marginally higher than the default (i.e. five) and within the recommended range [35]. Whilst this technique has been used for predictive purposes [16], it also has the ability to be used as a variable selection method [36]. This method was used as a variable selection technique to identify the prominent medical symptoms associated with depression within the key clusters [37]. A relative importance (or contribution) of each medical symptom variable for each of the key significant clusters was produced from the ML boosted regression. Higher values of relative importance for a medical symptom within a particular key cluster indicates a stronger relationships with depression in this cluster. This technique for variable reduction has been recognised as effective [39] and previously used to delineate lifestyle clusters associated with depression [18].

Those medical symptoms explaining at least 80% of the total log likelihood variation across clusters were used to identify the most important medical symptoms for explaining differences across clusters. Resultant medical symptoms were then grouped into the 17 broad medical categories.

The SOMs and hierarchical clustering were performed in R with the SOM using the Kohonen package [13]. The boosted regression and binary logistic regression statistical procedures were performed using Stata V14 software (StataCorp., 2014), with a Stata plugin for the boosted regression component of the analysis [38].

## Results

A summary of the results from the testing is presented in Fig 1.

### SOM Clusters

**Fig 2:**
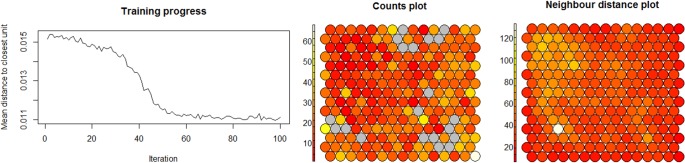
Training progress and SOM plots. Note: The “Training progress” graph indicates as the SOM training iterations distance from each node's weights to the samples represented by that node reduces and plateaus to indicate no more iterations were required. The “Counts plots” indicates reasonable samples were mapped to each node on the map. The “Neighbour distance plot” or U-Matrix indicates the distance between each node and its neighbours.

The distance from each node’s weights to the sample of people represented by that node was reduced to a minimum plateau as the SOM training iterations progressed, indicating that no more iterations were required (Fig 2). Taking into account the heterogeneous nature of the self-reported medical symptom data, the counts plot indicated a reasonable distribution of people numbers across the map. The neighbour distance plot indicated the distances between each node and its neighbours were mostly similar with only a few dissimilar nodes, later identified as outlying clusters (Fig 2).

**Fig 3:**
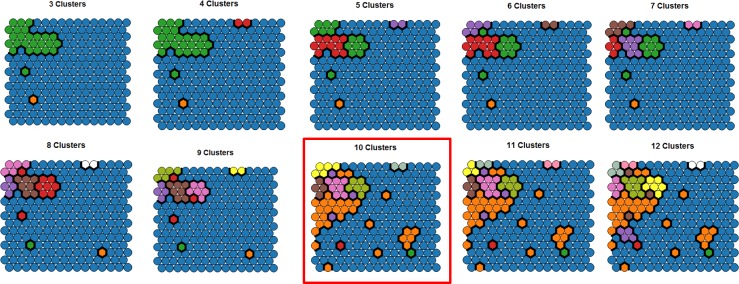
Hierarchical Cluster Options for SOM. Note: Clusters 3 to 12 solutions mapped onto the SOM grid. Colours indicate different clusters. The final 10 cluster solution selected for further analysis has been highlighted with a red border.

Three to 12 cluster solutions were considered (Fig 3) and the 10 cluster solution was selected for further investigation because of clear cluster differences in terms of depression rates. There were some isolated nodes in this cluster solution, later confirmed as outliers.

The final 10 cluster solution contained two dominant clusters (Table 1). One cluster was dropped from further analysis due to very low frequency (n = 8), leaving 9 of the 10 clusters for further analysis.

**Table 1:** Frequency Distribution of Initial Depression Ordered SOM Cluster Solution.

Cluster	Frequency	Percent
**1**	**3,108**	**79.25**
2	34	0.87
3	57	1.45
**4**	**446**	**11.37**
5	50	1.27
6	83	2.12
7	55	1.4
8	52	1.33
9	29	0.74
10 (Dropped)	8	0.2
**Total**	**3,922**	**100**

*Note*: Dominant clusters in bold. Cluster shaded dropped due to very small base (n = 8).

### Cluster validation

**Fig 4:**
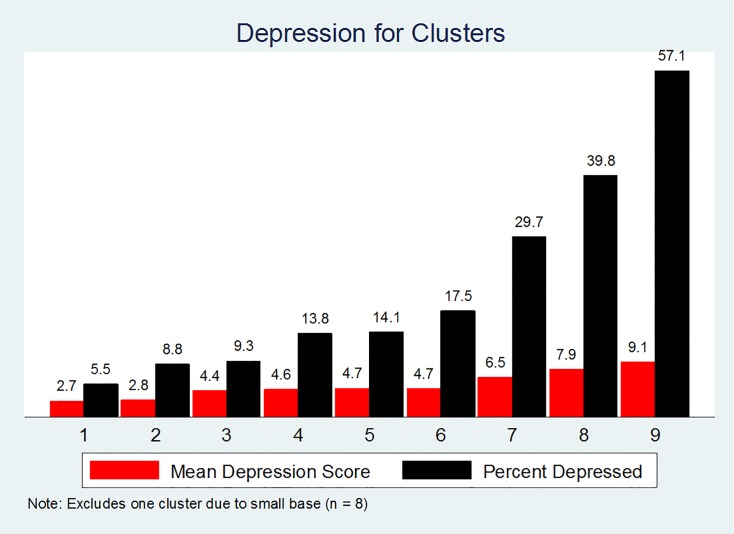
Mean depression scores and percent depression across final depression clusters. Note: “Mean Depression Score” is the average total PHQ-9 score which ranged from 0 to 27. “Percent Depressed” based on a total PHQ-9 ≥ 10.

Initial investigation into the relationship between the remaining nine participant clusters and the depression measures revealed that the clusters exhibited an order with respect to both the percentage of participants depressed within each cluster and the average depression score (Fig 4).

An initial inspection of the socio-demographics for the nine clusters (Table 2) showed clear differences. Due to the small frequencies for many of the clusters, only a qualitative investigation of socio-demographic differences was performed. Cluster 1 (n = 3,108) exhibited socio-demographics closest to the total across all cluster participants. Cluster 2 (n = 34) consisted of mostly male, non-Hispanic white with a high family income poverty ratio [40,41] and who were less likely to have never married. Cluster 3 (n = 57) consisted mostly of male, non-Hispanic white, older, married / with a partner, a household size of around two people, and a low family income poverty ratio. Cluster 4 (n = 446) members were more likely to be female, non-Hispanic white, middle aged, with a low family income poverty ratio, and less likely to have never been married. Cluster 5 (n = 50) were more likely to be older, non-Hispanic black, with a low family income poverty ratio, and less likely to never have been married. Cluster 6 (n = 83) members were more likely to be male, older, less than three members in the household, non-Hispanic white, and of low to mid family income poverty ratio, and less likely to have never been married. Cluster 7 (n = 55) were more likely to be middle aged, Mexican / Hispanic, with a low family income poverty ratio and less likely to have never been married. Cluster 8 (n = 52) were more likely to be female, older, non-Hispanic white, around two members in the household, with low family income poverty ratio and less likely to have been married. Cluster 9 (n = 29) were more likely to be young Mexican / Hispanic, with a large household and low family income poverty ratio.

**Table 2:** Demographic Profile Across SOM Clusters.

CLUSTER	Total	1	2	3	4	5	6	7	8	9
Sample (n)*	3,914	3,108	34	57	446	50	83	55	52	29
**Demographics**										
**Gender:**										
Male	49.6%	50.8%	60.4%	60.2%	38.9%	43.1%	63.9%	41.2%	31.2%	57.4%
Female	50.4%	49.2%	39.6%	39.8%	61.1%	56.9%	36.1%	58.8%	68.8%	42.6%
**Mean age** (years)	42.44	42.08	48.17	53.05	46.09	51.60	56.24	44.30	55.93	38.67
**Marital status:**										
Never	19.3%	20.4%	15.0%	11.6%	15.8%	14.6%	7.8%	14.7%	7.6%	19.6%
Married/Partner	65.3%	65.5%	66.4%	76.6%	64.7%	65.2%	63.9%	66.8%	48.9%	63.6%
Widowed/Divorced/Separated	15.4%	14.1%	18.6%	11.8%	19.6%	20.3%	28.4%	18.6%	43.5%	16.8%
**Mean household size**	3.22	3.23	3.19	2.23	3.07	2.95	2.64	3.26	2.55	3.94
**Mean family size**	3.02	3.03	3.07	2.94	2.91	2.75	2.49	2.94	2.39	3.88
**Race:**										
Mexican/Hispanic	14.3%	14.4%	6.0%	17.3%	15.3%	13.3%	5.6%	27.0%	2.6%	45.5%
Non-Hispanic white	67.5%	67.6%	78.4%	66.3%	67.3%	54.0%	74.2%	55.4%	81.3%	26.0%
Non-Hispanic black	11.4%	10.9%	15.6%	5.9%	12.8%	27.0%	17.4%	14.6%	10.1%	16.5%
Other	6.7%	7.1%	0.0%	10.4%	4.6%	5.7%	2.8%	2.9%	6.1%	12.0%
Family income poverty ratio**:										
Low	31.0%	29.2%	29.9%	39.8%	35.7%	42.6%	32.7%	64.9%	58.9%	68.8%
Middle	24.0%	24.5%	10.5%	19.0%	22.9%	23.4%	31.3%	15.9%	21.5%	11.2%
High	45.0%	46.4%	59.6%	41.2%	41.5%	33.9%	36.0%	19.2%	19.6%	20.0%
**Mean family income poverty ratio:**										
(Note: 1 = poverty line)	3.03	3.16	3.39	2.80	2.89	2.68	2.91	1.82	2.09	1.68

*Note*: Figures quoted take account of the survey design of NHANES with 15 strata, 31Primary Sampling Units (PSU).

*Total sample size varies per demographic as base includes all those with a depression score and valid answer given for demographic.

**Family income poverty ratio represents the ratio of family or unrelated individual income to their appropriate poverty threshold where groupings are based on eligibility for Special Supplemental Nutrition Program for Women, Infants, and Children (WIC): Low = 0.00–1.85 family income poverty ratio, Middle = >1.85–3.50 family income poverty ratio, and High = >3.50 and above family income poverty ratio.

### Identification of key clusters with higher depression rates

**Fig 5:**
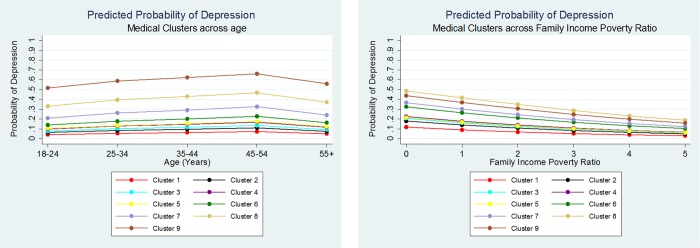
Predicted probability of depression across age and family income poverty ratio for each cluster.

The final binary logistic regression with depression as the outcome took into account the complex survey data of NHANES, as well as non-linearity, interactions and potential confounders (Table 3). The test for goodness of fit were not significant for the model indicating a good fit to the data (F(9,8) = 1.77, p = 0.216) [42]. Clusters 4 and 6 to 9 had significantly higher rates of depression than cluster 1 after controlling for the potential socio-demographic confounders. These five clusters were considered the key clusters for further analysis. Since the odds ratios for depression for clusters 2, 3 did not significantly differ from cluster 1 these clusters were excluded from future analysis. A significant interaction was found between the cluster with the highest rate of depression (cluster 9) and the family income poverty ratio (p = 0.036) (Fig 5). Thus, the relationship between the probability of depression and cluster 9 varied depending upon the rate of the family poverty income ratio.

**Table 3:** Binary Logistic Regression Model Odds Ratios with 95% Confidence Intervals.

Depression	OR	p-value	95% CI Low	95% CI High
*Cluster 1 (reference)*	1.00			
Cluster 2	1.67	0.341	0.55	5.04
Cluster 3	1.98	0.151	0.76	5.20
**Cluster 4**	2.24	**<0.001**	1.56	3.24
Cluster 5	2.10	0.180	0.68	6.43
**Cluster 6**	3.78	**<0.001**	2.17	6.57
**Cluster 7**	4.61	**<0.001**	2.21	9.63
**Cluster 8**	7.80	**0.001**	2.86	21.33
**Cluster 9**	6.33	**0.010**	1.67	24.02
**Cluster 9 X Family income poverty ratio**	2.00	**0.036**	1.05	3.81
**Gender**				
*Male (reference)*	1.00			
Female	1.86	**0.002**	1.31	2.64
**Age group**				
*18–24 years (reference)*	1.00			
25–34	1.37	0.326	0.71	2.63
35–44	1.61	0.177	0.79	3.29
45–54	1.92	**0.023**	1.11	3.34
55+	1.22	0.545	0.62	2.39
**Marital status**				
*Never married (reference)*	1.00			
Married/living with partner	0.54	**0.007**	0.35	0.82
Widowed/Divorced/Separated	0.79	0.172	0.55	1.12
Gender				
**Race**				
*Non-Hispanic white (reference)*	1.00			
Mexican American / Hispanic	0.88	0.368	0.67	1.17
Non-Hispanic Black	1.17	0.436	0.77	1.76
Other	0.77	0.391	0.42	1.43
**Education**				
*Grades 11 and below (reference)*	1.00			
High School / GED Equivalent	0.43	**0.039**	0.20	0.95
Some College / AA / College or Above	0.59	**0.008**	0.40	0.85
**Family income poverty ratio**	0.60	**0.002**	0.45	0.80
**Education X Family income poverty ratio**				
*Grades 11 and below (reference)*	1.00			
High School / GED Equivalent	1.43	0.073	0.96	2.11
Some College / AA / College or Above	1.24	0.089	0.96	1.58
*Constant*	0.16	<0.001	0.08	0.34

*Note*: OR = Odds Ratio, CI = Confidence Interval. Multivariate logistic model taking account of complex survey methodology (N = 3,584, 15 Strata, 32 PSUs). Bold p-values indicate significant p<0·05. Cluster 9 OR = 12.67 (95% CI: 1.75, 91.56) taking into account the interaction.

### Medical symptoms most prominent within key clusters

**Fig 6:**
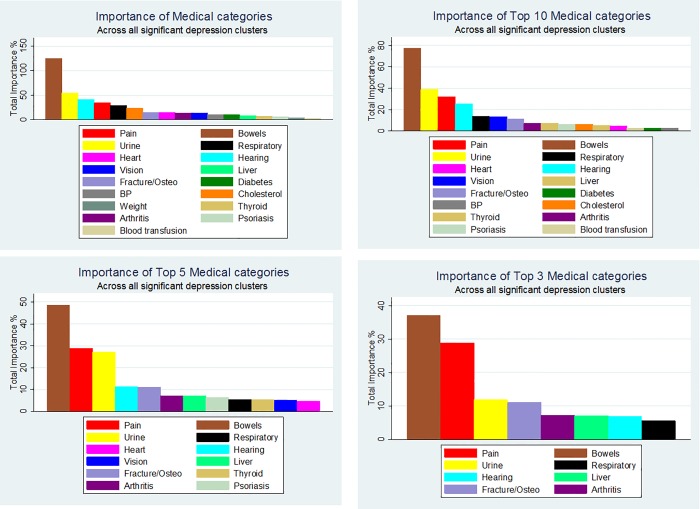
Importance of medical categories that make up the key significant clusters. Note: Based on total boosted relative importance percentage across all clusters. Summed percentage from boosted regression across all five key significant clusters, thus total >100%.

ML boosted regression was used to establish which medical symptoms were associated with depression for each of the five key significant clusters. The top medical symptom variables explaining approximately 80% of the total log likelihood for each cluster were selected for categorisation and further investigation. Bowel symptoms (e.g. bowel movements per week, stool type) dominated the relative importance percentage across all the five key clusters (Fig 6). Further investigation into the top 3 to 10 ranked medical categories from the ML boosted regression found that bowel, pain and urine symptoms consistently exhibiting a relatively high importance percentage for each of the key clusters.

**Fig 7:**
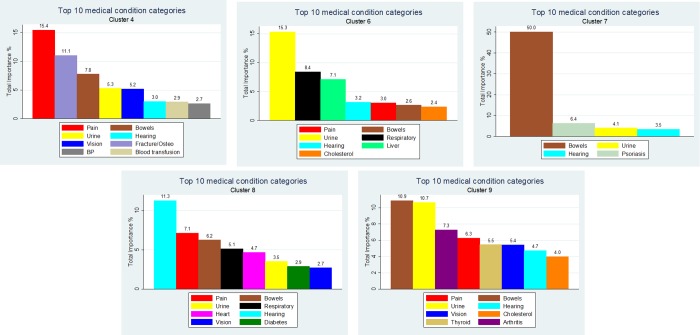
Total percentage importance of medical conditions for each key significant cluster. Note: Clusters presented in order of percent depressed. Note: Percentage sum does not take account of direction of relationship.

The top 10 key medical symptom categories for the five key significant clusters indicated that each cluster exhibited different medical symptoms (Fig 7). However, bowel symptoms were consistently included in the highest ranked medical symptoms across all five significant depressive key clusters. In addition, the bowel symptoms dominated for cluster 7 and cluster 9, and had relatively high importance (i.e. >5%) for four of the five key clusters. Pain symptoms had the highest relative importance in cluster 4 and urine symptoms had relatively high importance (i.e. <10%) for two of the five key clusters. Whilst hearing symptoms were important in all five of the key clusters, they only dominated in cluster 8.

The individual clusters showed clear delineation with respect to medical conditions. The top three medical symptoms for cluster 4 related to the skeletal symptoms of pain, fractures and osetoporosis, and bowel symptoms. Cluster 6 was dominated by urinary medical symptoms. Cluster 7 was clearly dominated by bowel medical symptoms. Cluster 8 was a generally unwell cluster with the top five medical symptoms related to hearing, pain, bowels, respiratory and heart. Finally the top two medical symptoms for cluster 9 related to bowels and urine.

## Discussion

Irrespective of country, research has consistently found a high level of comorbidity between specific (e.g. sleep, appetite) and nonspecific symptoms and depression [43,44] but it has been difficult to identify the key somatic symptoms most prominent in this condition. This study utilized two machine learning techniques, complemented by traditional binary logistic regression analyses, to detect complex interactions between large numbers of medical symptoms in order to identify those most strongly linked to depression in an atheoretical manner. ML techniques have been used in the area of big data informatics in mental health. For example, text analysis [45] and regression models [46] have been used to predict the risk of suicide from clinical notes, but these techniques have not previously been used to investigate the relationship between depression and medical symptoms using epidemiological community based population data. The visual simplification of complex medical symptom data into clusters, using SOM, allows the researcher to easily identify the strength of the similarities across the map. The ML SOM’s intention to mimic an artificial network that learns, without supervision, has proven effective in creating nodes, subsequently grouped into clusters identified by a standard hierarchical clustering. Nine clusters of participants based on medical symptoms were found using the unsupervised graphical SOM ML technique. Traditional binary logistic regression showed that five of the nine clusters were characterised by higher rates of depression after controlling for potential confounders and taking account of the complex survey methodology of the population data.

A boosted regression ML algorithm was used to provide a relative importance percentage for each medical symptom for each of the five key significant clusters, allowing the easy grouping of symptoms into medical categories. The ML boosted regression algorithm was able to untangle the array of medical symptoms and detect three key medical condition categories as being particularly related to depression: bowel, pain and urinary symptoms. Of these categories, bowel symptoms dominated, validating previous research regarding the high comorbidity between gut symptoms and IBS with common mental disorders, including depression [3,47].

Gut disorders in particular share links with depression. Irritable bowel syndrome (IBS) [3] has been found to be closely associated with mental health conditions. IBS is not only comorbid with psychiatric conditions, but also comorbid with non-gastrointestinal somatic disorders [48]. Crohn’s disease [49] and gastro-oesophageal reflux disease (GORD) [50] are similarly associated with higher rates of mood disorders than would be expected by chance. All these interrelationships impact on the quality of life, treatment compliance, length of stay in hospitals, costs of health care, morbidity and possibly mortality of individuals affected.

Medical symptoms relating to stool type and frequency and constipation were included in the bowel categorisation for this study, and these indicators have all been related to mood [51]. Recently, ML boosted regression has identified an association between the gastrointestinal biomarker of bilirubin with depression [36] and bilirubin has been linked to varying stool type based on the speed at which the intestinal contents travel through the bowel [52].

There is an increasing focus in medical research on the role of symbiotic gut microbiota in health and disease, including mental health. Indeed, the human gut microbiota, and what is termed the ‘gut-brain axis’, are now increasingly regarded as potentially critical drivers of mood and behaviour, with much of the biological dysregulation associated with depressive symptoms and the diagnosis of clinical depression influenced by the gut microbiota [53]. Such microbiota-influenced dysregulation involves inflammatory, metabolic, oxidative stress, HPA axis, neurotransmitter/neuropeptide, brain plasticity and other systems [54]. Moreover, the normal intestinal barrier function is compromised in depression [55]. This ‘leaky gut’ allows intestinal-microbe-derived lipopolysaccharide (LPS), an endotoxin, to gain access to the periphery. Even very low levels of LPS can provoke much of the aforementioned biological dysregulation noted in depression.

Importantly, many of the lifestyle and environmental factors connected to depression have a detrimental influence on the composition of the normal human microbiota. As just one example, unhealthy dietary patterns that increase the risk for depression [56] also diminish microbial diversity [57]. Long-term, habitual diets are one of the strongest influences on gut microbial composition, determining an individual “enterotype” [58], however dietary change can prompt change in gut microbiota composition within 24 hours [59]. The consumption of complex carbohydrates, plant-based foods/fruits and vegetables [58,60] positively influences microbial composition, synthesis of anti-inflammatory short chain fatty acids, and host health. Conversely, high fat diets trigger microbial dysbiosis, intestinal permeability (‘leaky gut’) and inflammation [61]. We have previously demonstrated that healthy dietary patterns are associated with a reduced likelihood of depressive symptoms in adults participating in the NHANES [62]. This suggests that unhealthy dietary behaviors may be a key factor negatively influencing both gut health and depression, with bowel symptoms signifying poor gut health.

### Strengths and Limitations

The strengths of this study lie in the benefits of using both unsupervised and supervised ML techniques to identify patterns in data, using a large number of heterogeneous self-reported medical symptoms to form five clusters of individuals with relatively high rates of depression, most likely to have remained hidden using traditional statistical techniques. The largest cluster of participants (cluster 4, n = 446) comprised 7% moderately and 7% severely depressed participants; this compares to rates of 5% and 3% respectively in the general 2009 to 2010 US population in NHANES. The remaining key clusters (6 to 9) consisted of smaller groups of participants, with 15% moderately and 14% severely depressed participants overall. A main limitation with this study is the cross-sectional nature of the NHANES data that restricts the ability to infer causality. However, the use of this community population based survey data has the advantage of being representative of the large US population sampled during 2009 to 2010. The large number of participants included in this study, with its rigorous complex survey sampling methodology, ensures the data possess a good description of the relative characteristics of the civilian noninstitutionalised US population. As compared to other methods of data gathering, surveys are able to extract data that closely mirror attributes of the larger population.

It is acknowledged that the PHQ-9 instrument relates to depressive symptoms, and does not represent a clinical diagnosis of depression. Thus, this self-report instrument may have missed less severe cases of depression [27,28] exaggerating the imbalance in the data. Furthermore, the depression symptoms picked up by the PHQ-9 instrument for this study, such as fatigue, psychomotor problems, or insomnia are symptoms very common in medical conditions. Thus, it was not surprising that the results from this study confirmed prior research identifying depressive symptoms being often elevated in people with medical symptoms [63]. The relationship between medical symptoms and depression is complex and often bidirectional. However, the identification of the dominant medical symptoms, such as those of the bowel cluster in this study, may be used to improve screening tools for depression in medically ill patients and to shed light on possible pathogenic processes. It is acknowledged that individuals with depression are more likely to report somatic conditions, and IBS has been found to be a disorder with a psychosomatic aspect [47]. However, the NHANES study is considered representative of the US noninstitutionalised civilian population and has been used to produce health statistics for the US and in many studies investigating depression (e.g. to examine the prevalence, treatment and control of depressive symptoms [64]).

We addressed the limitation of the imbalance in the data of having only approximately 8% of the sample classified with depression by including only those clusters with high depression rates, hence reducing the impact of this imbalance on our analysis.

There are potential limitations in using the proposed ML techniques. The SOM can become conceptually expensive as the number of variables and the grid size increases, causing the number of distances the algorithm needs to compute to increase exponentially. In addition, the SOM requires a value for each variable for each participant in order to generate a map, so missing data poses issues for map generation with SOMs. Alternative less computer intensive traditional statistical techniques, such as k-means clustering or latent class analysis, could have been used. However, the SOM algorithm has been found to provide better results than either of these methods in the case of large data sets [65–67] such as used in this study.

The ML boosted regression has the advantage of automatically incorporating interaction effects when evaluating variable importance which is not possible with traditional statistical regression modelling [37]. Also, variable selection processes, such as stepwise or regularized regression make variable selection difficult when there are highly correlated predictors as is the case with medical symptoms. The boosted regression overcomes this problem by reducing the number of selected variables at each iteration thereby being able to deal with highly correlated variables. However, ML boosted regression can fail to perform well with small data sets [68]. In addition, the training process can be computationally memory intensive due to the fact that trees are built sequentially, requiring advanced computing capability such as parallel processing. In addition, the regularization implemented to reduce the effects of overfitting can mean the optimal number of iterations for a suitable shrinkage parameter can be considerably large [69].

Whilst this study performed validation using a random split of data into 60% training and 40% validation at the ML boosted regression stage, no validation of the methodology was performed on a separate data set using self-reported medical symptom data. However, this methodology has been successfully implemented to identify lifestyle clusters associated with depression [70].

## Conclusion

This study implemented two ML algorithms and a standard binary logistic regression to identify and describe clusters of individuals with higher rates of depression based on self-reported medical symptoms in a large, cross-sectional epidemiological community based population study. Bowel symptoms, covering bowel frequency and stool type, were identified as the predominant concurrent symptom category for the key clusters with a significant positive relationship with depression across 17 varied medical symptom categories. This study encourages the future use of machine learning techniques to compliment traditional statistical approaches in the analysis of epidemiological studies to assist clinicians detect potential latent associations that can be further refined and clarified. This study also supports a research focus on the potential importance of the bowel symptoms, the gut and its resident microbiota in mental health research.

## Abbreviations

DIPIT: Data Integration Protocol In Ten-Steps
ML: Machine-learning
MART: Multiple Additive Regression Trees
NCHS: National Center for Health Statistics
NHANES: National Health and Nutrition Examination Survey
PHQ-9: Patient Health Questonnaire-9
SOMs: Self-organizing maps
